# Supplementary material for: Causal Relationship between Aging and Anorexia Nervosa: A White-Matter-Microstructure-Mediated Mendelian Randomization Analysis
Source: Biomedicines. 2024 Aug 16;12(8):1874. doi: 10.3390/biomedicines12081874 (PMC11351342; doi:10.3390/biomedicines12081874)
Supplement: Supplementary file 1 [file biomedicines-12-01874-s001.zip › Supplementary Figure/Figure S4/Singe SNP plot of MR analysis for TL to ubm-b-1897.pdf]

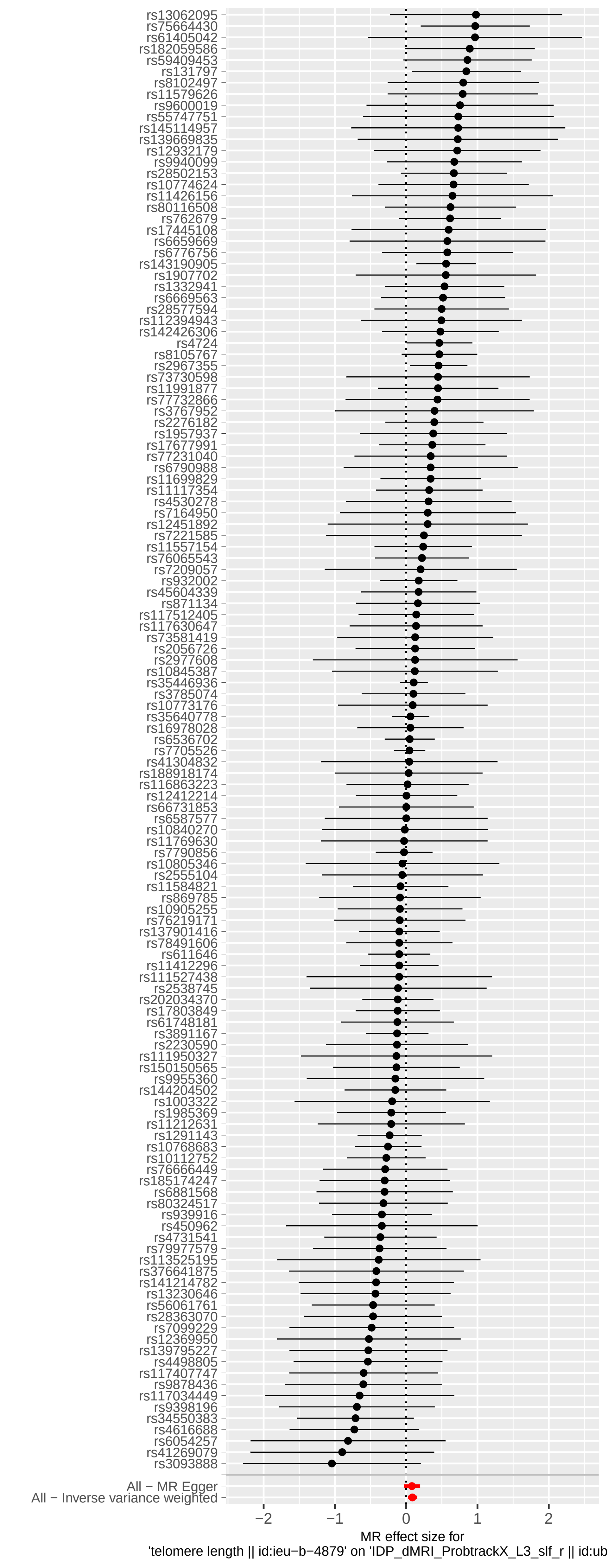
rs13062095
rs75664430
rs61405042
rs182059586
rs59409453
rs131797
rs8102497
rs11579626
rs9600019
rs55747751
rs145114957
rs139669835
rs12932179
rs9940099
rs28502153
rs10774624
rs11426156
rs80116508
rs762679
rs17445108
rs6659669
rs6776756
rs143190905
rs1907702
rs1332941
rs6669563
rs28577594
rs112394943
rs142426306
rs4724
rs8105767
rs2967355
rs73730598
rs11991877
rs77732866
rs3767952
rs2276182
rs1957937
rs17677991
rs77231040
rs6790988
rs11699829
rs11117354
rs4530278
rs7164950
rs12451892
rs7221585
rs11557154
rs76065543
rs7209057
rs932002
rs45604339
rs871134
rs117512405
rs117630647
rs73581419
rs2056726
rs2977608
rs10845387
rs35446936
rs3785074
rs10773176
rs35640778
rs16978028
rs6536702
rs7705526
rs41304832
rs188918174
rs116863223
rs12412214
rs66731853
rs6587577
rs10840270
rs11769630
rs7790856
rs10805346
rs2555104
rs11584821
rs869785
rs10905255
rs76219171
rs137901416
rs78491606
rs611646
rs11412296
rs111527438
rs2538745
rs202034370
rs17803849
rs61748181
rs3891167
rs2230590
rs111950327
rs150150565
rs9955360
rs144204502
rs1003322
rs1985369
rs11212631
rs1291143
rs10768683
rs10112752
rs76666449
rs185174247
rs6881568
rs80324517
rs939916
rs450962
rs4731541
rs79977579
rs113525195
rs376641875
rs141214782
rs13230646
rs56061761
rs28363070
rs7099229
rs12369950
rs139795227
rs4498805
rs117407747
rs9878436
rs117034449
rs9398196
rs34550383
rs4616688
rs6054257
rs41269079
rs3093888
All − MR Egger
All − Inverse variance weighted
−2
−1
0
1
2
MR effect size for
'telomere length || id:ieu−b−4879' on 'IDP_dMRI_ProbtrackX_L3_slf_r || id:ub
